# Additional accommodative controls in near heterophoria targets do not improve accommodative responses in young adults

**DOI:** 10.1111/opo.13476

**Published:** 2025-02-25

**Authors:** John Siderov, Baskar Theagarayan, Muneebah Zahir, Niall J. Hynes

**Affiliations:** ^1^ Centre for Vision Across the Life Span, School of Applied Sciences University of Huddersfield Huddersfield UK

**Keywords:** accommodation, esophoria, exophoria, heterophoria, test–retest repeatability

## Abstract

**Purpose:**

This study evaluated newly designed tests to measure near heterophoria, comparing them to the Maddox Wing and Howell card, and assessed whether accommodative responses differed between the different heterophoria test targets.

**Methods:**

Near horizontal heterophoria of 20 visually normal young adults was measured using the Maddox Wing, Howell Card and two versions of a newly designed Huddersfield Heterophoria Test (HHT) card. The HHT cards are based on the Prentice method, either with or without an additional spatially defined grating superimposed on the scale. The addition of a spatially defined grating has been suggested to control accommodation better. A single examiner was used to take measurements on two separate occasions, presenting each test in a random order. Monocular accommodative responses to each heterophoria target were also obtained in a separate session by another examiner.

**Results:**

Heterophoria measurements between the four tests were not significantly different, neither clinically nor statistically. Differences in test–retest measurements between test and retest conditions were small and not significantly different. Mean accommodative responses were also not significantly different between the test targets.

**Conclusions:**

Differences in target configurations in the Maddox Wing, Howell card or new HHT cards did not influence accommodative responses in a sample of young adults. The newly designed HHT cards (or other variations of the Prentice method) may be used to measure near horizontal heterophoria in a young adult population either with or without additional horizontal lines superimposed on the scale.

**Key points**

- Mean near heterophoria measurements obtained using the Maddox Wing, Howell card and the newly designed Huddersfield Heterophoria Test cards did not differ significantly.
- Accommodative responses between the different heterophoria targets were not significantly different, indicating that additional target features aimed at controlling accommodation are redundant.
- Test–retest repeatability of a new version of the Prentice card method was comparable to other commonly used heterophoria measurement methods.

## INTRODUCTION

Measurements of heterophoria are commonly used in routine optometric examinations to facilitate an evaluation of a patient's binocular visual status. Heterophorias present as latent misalignments of the visual axes in the absence of fusion and can occur in the horizontal, vertical and even, albeit less frequently, cyclorotational meridians.^1^, ^2^ They may be associated with symptoms of visual discomfort, suggesting poor compensation of the heterophoria, and as such, their measurement and evaluation have long been recommended.^1^, ^3^, ^4^

Several different methods to assess heterophoria are available and include the cover test, von Graefe's method, Maddox Rod and Maddox Wing (for near), and Thorington's method and its variations.^1^, ^5^ The methods vary in the targets used and how binocular dissociation is obtained. Nevertheless, despite differences in the methods, a common requirement when measuring the near horizontal heterophoria is good control of accommodation.^4^, ^6^, ^7^, ^8^, ^9^ Unreliable or unsteady accommodation in patients may cause changes in the vergence position (through the AC/A ratio), presumably leading to variations in the measured heterophoria.

However, to the authors' knowledge, direct assessment of accommodation while measuring horizontal heterophorias has not been reported. Rather, evidence for the influence of accommodation comes from indirect observations. In a study investigating enhancements to the Maddox Wing tangent scale, Pointer^7^ investigated how differences in target construction could affect heterophoria measurements. He created alternate tangent scale targets incorporating reduced type sizes of 4 and 10 point, and superimposed horizontal square wave gratings (effectively two parallel bars) matched to the fonts, of 1.67 and 0.67 cycles per degree (cpd), respectively. The premise for using square wave gratings came from studies showing that the accommodative response was optimal for targets comprising relatively low spatial frequencies.^10^, ^11^, ^12^, ^13^ Pointer's results showed that the target with the smallest font and associated square wave grating produced the most reliable heterophoria measurements, resulting in the best repeatability, albeit conceding that the differences between the different targets were small.^7^ Pointer concluded that, although using smaller target sizes with the additional square wave grating may improve the repeatability of heterophoria measurements, differences are likely to be small and potentially of little clinical significance, particularly in adults.^7^

Wong and colleagues^9^ reported results using a new type of heterophoria card based on a design described originally by Prentice^14^ (sometimes referred as the Thorington method^15^). The original Prentice card comprised a horizontal printed scale with capital letters on the left and numerals on the right, separated by a vertical arrow pointing downwards. Each letter or number represented one prism dioptre. Dissociation was achieved using a vertical prism.^14^ The Howell phoria card^9^ is a modification of the Prentice card and includes, for the reasons given by Pointer,^7^ a square wave grating pattern of 2.5 cpd superimposed on the recording scale, an oval shape to reduce fusional cues and a colour‐coded number scale to ease identification of the presence and magnitude of the heterophoria.^5^ The Howell card was designed to be used in free space and is easily administered to both children^16^, ^17^ and adults. It shows good repeatability compared with other techniques.^9^ As such, this Prentice‐based method^14^ has several advantages including ease of administration, ability to be used in free space or with a phoropter and portability. Although good control of accommodation is suggested with the Howell card,^9^ this has not been tested.

Modification of near heterophoria targets by adding a spatially defined square wave grating to the heterophoria scale is appealing,^7^, ^9^ given evidence on the responsiveness of accommodation to such stimuli.^10^, ^11^, ^12^, ^13^ Nevertheless, whether such a modification contributes to better accommodative control has not been tested directly. In view of the reported importance of controlling accommodation when assessing horizontal heterophoria and the potential for enhancing accommodative accuracy, the aim of the current study was to compare four different subjective tests of heterophoria, namely the Maddox Wing, Howell card and two new test designs based on the principles of the Prentice card, one with and the other without the addition of a square wave grating (Huddersfield Heterophoria Test or HHT). The mean differences in heterophoria between each test and test–retest repeatability were compared. Additionally, monocular accommodative responses to each heterophoria target were also measured objectively to determine if the different target configurations produced differences in accommodative response.

## METHODS

### Participants

Consenting adults from the university's students and staff were invited to participate in the study. The sample size for the study comprised 20 subjects (11 males and nine females) with a mean (±SD) age of 21.6 ± 0.9 years. Participants were pre‐screened to exclude strabismus or amblyopia, had a visual acuity of 0.00 logMAR (6/6) or better in each eye and stereopsis of at least 60″. Normal amplitude of accommodation was assumed. Where required, refractive errors were corrected using contact lenses to achieve the required visual acuity. There were 11 myopes (≤−0.50D), one hypermetrope (≥+1.00D) and eight emmetropes (−0.25D to +0.75D). The sample size was sufficient to obtain a power of 80% at the 5% level (two‐tailed) for an effect size of 1 prism dioptre (Δ) difference between the tests. The study followed the tenets of the Declaration of Helsinki, and approval of the study protocol was obtained from Huddersfield School of Applied Sciences Research Integrity and Ethics committee. Written informed consent was obtained after the procedures were explained to participants and before the start of data collection.

### Heterophoria tests

**FIGURE 1:**
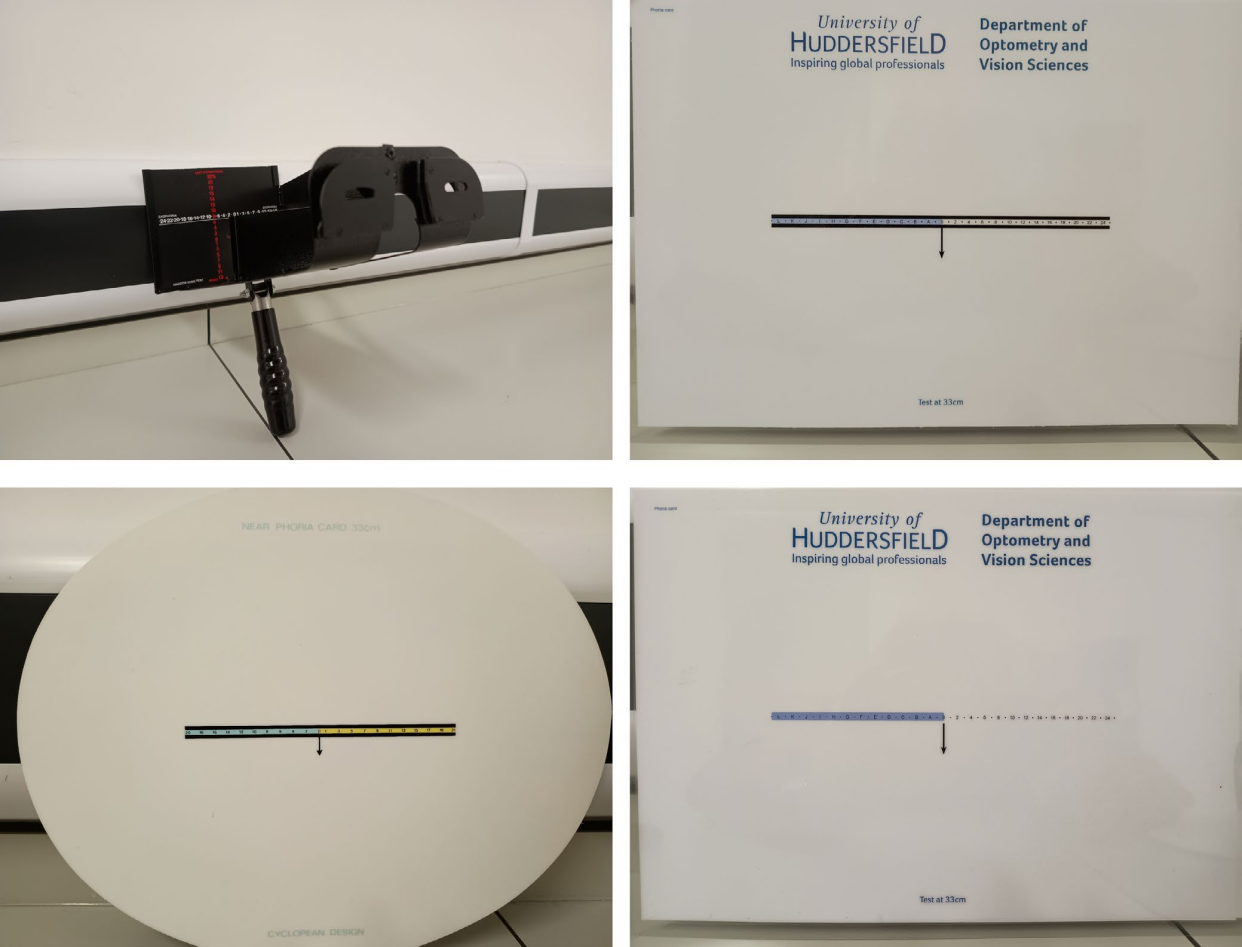
Image of the four near heterophoria tests used in the study. Maddox Wing (top left) and the Howell card (bottom left) and the Huddersfield Heterophoria Test (HHT) cards (right panels), with (top) and without (bottom) the horizontal bars.

The Maddox Wing (Maddox Wing Test, Keeler UK, keeler.co.uk/maddox‐wing‐test.html) is a hand‐held instrument commonly used in the UK^7^ to assess near heterophoria at a fixed distance of 30 cm (Figure 1). For this study, only horizontal heterophoria was assessed, although vertical and cyclophoria measurements are also possible. Participants hold the instrument and view a numbered measurement scale and recording arrow printed on to a card mounted at the end of the instrument through fixed apertures. The scale indicates exo‐ or esophoria in Δ. Binocular dissociation is achieved with a physical septum, so the observer sees the measurement scale with one eye and the recording arrow with the other. Participants are asked to indicate the relative position of the recording arrow relative to the scale and verbally respond with the appropriate number.

The Howell phoria card (sussex‐vision.co.uk/howell‐phoria‐neardistance‐pair) is an oval plastic card, approximately 30 × 25 cm in diameter, that is used to measure horizontal heterophoria (Figure 1).^5^, ^9^ Printed in the centre of the card is a numbered tangent scale, shaded with different colours to the right (yellow) and left (blue) of the zero point. A short vertical arrow, positioned just below the zero point, acts as a measuring device. Both near (33 cm) and distance (3 m) cards are available, although only the near card was used in this study. A vertical prism is used to achieve dissociation. Typically, a 6 Δ prism, placed base down before the observer's right eye, is sufficient to produce adequate dissociation, although, if required, slightly higher powers may be used without detriment.^18^ Once dissociated, subjects see two lines of numbers and arrows (one with each eye) and report the position of the ‘top’ arrow relative to the lower scale. The original Prentice (sometimes referred to as Thorington) method utilised a row of letters on one side and numbers on the other side, separated by a vertical arrow.^14^, ^15^ To aid the examiner, the Howell card added both colour coding and odd and even numbers to indicate exo‐ or esophoria (with a base down prism in front of the right eye, the blue even and yellow odd numbers indicate exophoria and esophoria, respectively).^9^ The scale, calibrated in Δ for the 33 cm test distance, is surrounded by two thick horizontal lines, which are included to act as an accommodative lock.^9^, ^15^ The thickness of the lines corresponds to a spatial frequency of approximately 2.5 cpd, thought to provide an optimal accommodative stimulus.^10^, ^11^, ^12^, ^13^

The newly devised Huddersfield Heterophoria Test (HHT) cards, developed by the first author, are also based on the original Prentice idea.^14^, ^15^ Two versions of the HHT cards were designed, one with the same thickness horizontal lines as found in the Howell card (HHT Lines) and one without (HHT No Lines) (Figure 1). The cards are rectangular in shape (approximately 20 × 28 cm) and comprise, in the centre, a printed tangent scale with a vertical arrow located in the middle. The left side of the scale was shaded (light blue) and comprised capital letters (from A through to L), while the right scale was unshaded and included even numbers from 2 through to 24, with each letter or number separated by a single point. The letters and numbers were the same size as in the Howell card, equivalent to about N6 font size, or a reduced Snellen fraction of approximately 6/15 at the 33 cm test distance. A zero (0) point divided the two sides and marked the position of the arrow. The cards were calibrated for a 33‐cm test distance so that each letter or number separation represented 2 Δ, with the small points between them being 1 Δ. A 6 Δ base down prism before the right eye was used to achieve binocular dissociation. Once dissociated, participants were asked to report the position of the ‘top’ arrow on the scale below, indicating whichever letter or number the arrow pointed towards. The letters and blue shading represented exophoria, while the numbers and no shading indicated esophoria.

For each test, participants were instructed to keep the target numbers or letters clear and maintain their attention on the respective recording arrows to maximise the stability of the heterophoria measurements.^19^

### Protocol

#### Heterophoria measurements

Measurements of heterophoria were performed by a single examiner following a standard protocol for each test. Near heterophoria was measured in a single session using the four tests (Maddox Wing, Howell card and the two HHT cards), presented in random order for each participant using a random number table. The tests were presented in a continuous fashion and flash presentations^9^ were not used. Participants were instructed to keep the target scales on each test as clear as possible and to report the position of the measurement arrow on the respective scales. Participants held the instruments at the correct test distances for each test, checked for each presentation. A trial frame was used to hold the dissociating prism for the Howell and HHT cards. Data collection occurred under photopic conditions in a well‐lit laboratory. A single heterophoria measure was obtained for each test once the procedure was explained to the participant (which took only a minute or two). Heterophoria measurements were repeated by the same examiner in separate sessions approximately 2 weeks after the first test session.

#### Accommodative measurements

Measurements of accommodative response were performed by a second examiner blind to the results of the heterophoria measurements. Accommodative responses were measured using a Shin‐Nippon NVISION‐K5001 infra‐red autorefractor (rexxam.co.jp/eye‐care/products/nvk5001.html). The zero points on each of the heterophoria targets were aligned with each participant's right eye using a holding clamp. The target scale from the Maddox Wing was removed from the instrument to facilitate recording of the accommodative response. Measurements were performed on the right eye under binocular viewing (i.e., left eye unoccluded). Participants were directed to look at the zero point on the respective heterophoria cards and to keep the target as clear as possible. Testing was performed at a fixed distance of 33 cm to ensure consistency between tests. To determine the accommodative responses, measurements of refractive error were also obtained in the distance. Participants were asked to fixate a high contrast Maltese cross target placed at 6 m in front of their right eyes. Five consecutive readings were taken for each condition, including the distance measure, through a single button press on the autorefractor. The mean spherical equivalent was calculated for each reading by summing the spherical result with half the cylinder value. The average of these measurements for each condition was then used to determine the accommodative response for each target (i.e., the difference between the distance and near values). As for the heterophoria measurements, where required, participants were corrected for any significant refractive error using contact lenses. Data collection occurred within 1 week following the second heterophoria measurement and under the same photopic conditions. One participant did not attend for the accommodative measurements, while data from a second participant could not be used due to incompleteness, resulting in 18 complete sets of measurements.

### Statistical analysis

A two‐way, repeated measures ANOVA was used to compare the heterophoria measurements between tests and between test and retest conditions. A separate one‐way repeated measures ANOVA was used to compare the mean accommodative measurements for each test. Where required, the levels of statistical significance included a Greenhouse–Geisser correction for departures from sphericity.^20^ Data analysis was completed using SPSS version 28.0 (ibm.com/products/spss‐statistics) and Microsoft Excel version 16 (microsoft.com/en‐gb/microsoft‐365/excel). Results were considered statistically significant for *p* < 0.05.

## RESULTS

**FIGURE 2:**
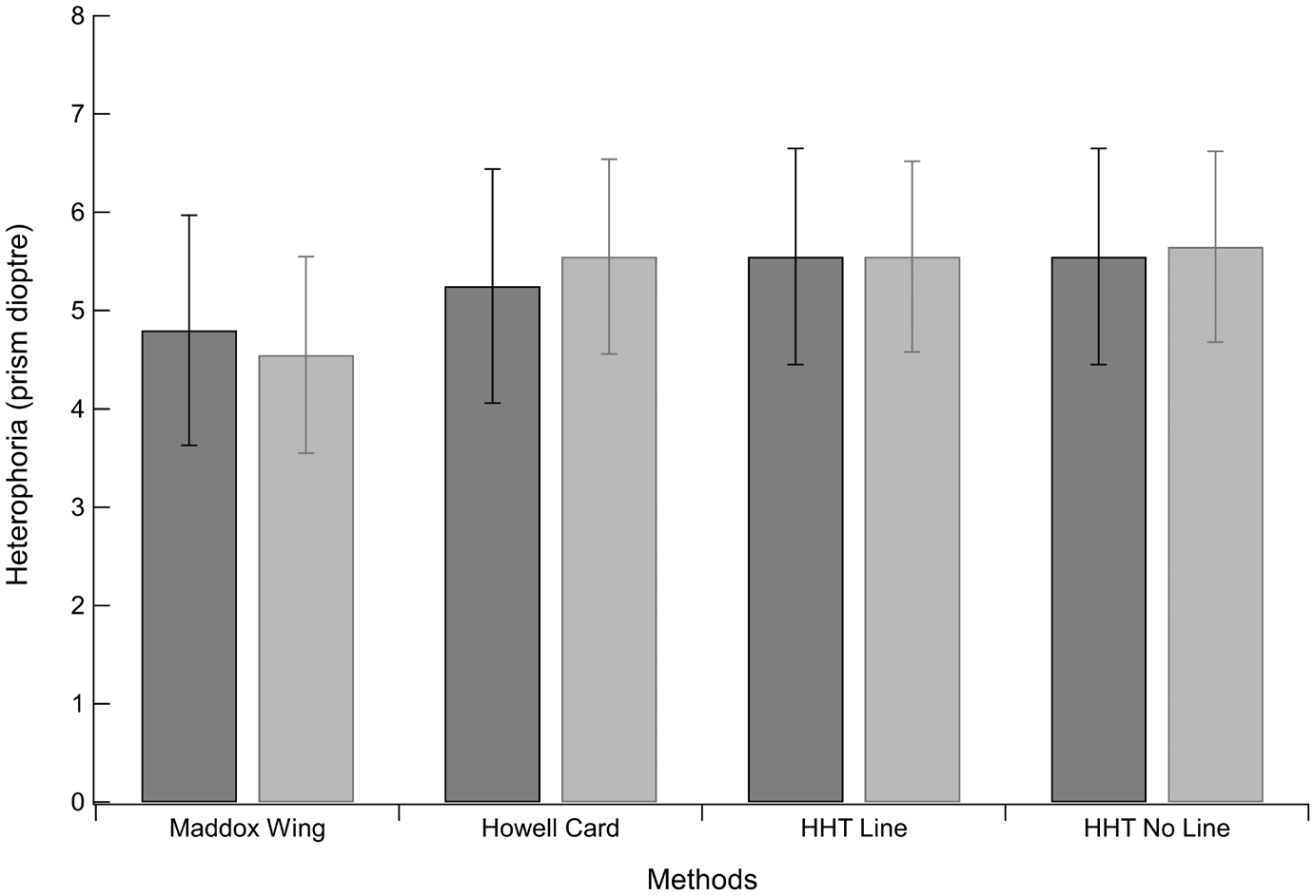
The mean heterophoria values (Δ exophoria) for each of the four tests are shown. The darker shaded bars represent the initial test measurements, and the lighter shaded bars represent the retest. Error bars represent ±1 SE. HHT, Huddersfield Heterophoria Test.

Heterophoria values ranged from 1 Δ esophoria to 16 Δ exophoria across the sample and tests used, although most participants had exophoria. Table 1 summarises the mean heterophoria measurements across all 20 participants, four different tests and test–retest conditions. For clarity, positive values indicate exophoria. Figure 2 shows these results in a histogram. The darker shaded bars represent the test condition and the lighter shaded bars the retest. There was a small difference, approximately <1 Δ, between the Maddox Wing results and findings using the other tests. Differences in heterophoria between the test and retest conditions were also small across the tests, generally <0.3 Δ.

**TABLE 1:** Mean test and retest heterophoria results (Δ) for each heterophoria test (averaged across 20 participants). Positive values indicate exophoria. The associated mean accommodative responses (dioptres, D) for each test target are also shown (averaged across 18 participants). The SE for each measurement is indicated in brackets. HHT, Huddersfield Heterophoria Test.

	Test (prism dioptres)	Retest (prism dioptres)	Accommodative response (D)
Maddox Wing	4.80 (1.17)	4.55 (1.00)	2.29 (0.12)
Howell card	5.25 (1.19)	5.55 (0.99)	2.26 (0.12)
HHT Lines	5.55 (1.10)	5.55 (0.97)	2.34 (0.09)
HHT No Lines	5.55 (1.10)	5.65 (0.97)	2.29 (0.12)

A two‐way repeated measures ANOVA was performed on the heterophoria measurements, with test type and test session as the main factors.^20^ The ANOVA showed no significant main effects either for test type [*F* (1.46, 15.15) = 2.72, *p* = 0.10] or test session [*F* (1, 0.056) = 0.02, *p* = 0.89], nor was the interaction significant [*F* (1.44, 1.09) = 0.28, *p* = 0.68].

**FIGURE 3:**
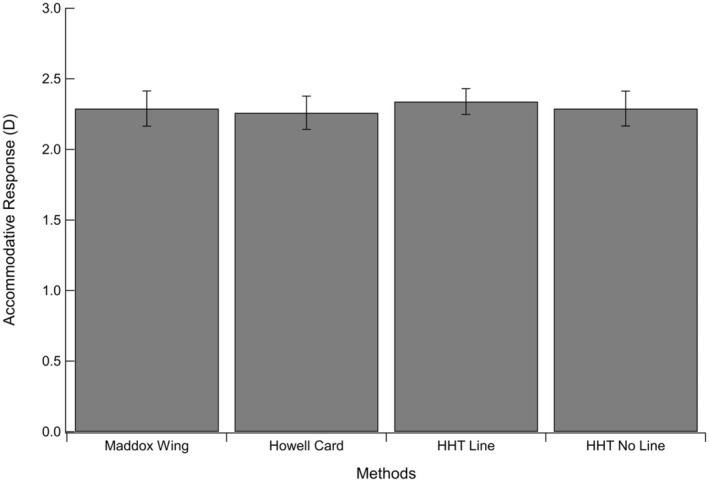
The mean accommodative responses (right eye only) as a function of the four heterophoria test targets are shown. Error bars represent ±1 SE. HHT, Huddersfield Heterophoria Test.

Table 1 shows the associated mean accommodative responses (in dioptres and averaged across 18 participants) for each heterophoria target. Differences in accommodative responses between the four heterophoria targets were small. These results are also shown in Figure 3, where accommodative responses (in dioptres) are plotted as a function of each heterophoria test. A one‐way, repeated measures ANOVA confirmed no significant difference in the accommodative response as a function of the test target [*F* (2.85, 0.01) = 0.09, *p* = 0.96].

## DISCUSSION

### Heterophoria

All but one participant showed exophoria at near, irrespective of the test used. Despite the relatively small sample size used in the study, this result is not surprising, as it is consistent with long‐established norms for adult subjects showing mean exophoria at near.^4^, ^21^, ^22^ A more pertinent outcome of the current study is that the heterophoria measurements between the different tests and the test–retest conditions were minimal and neither clinically nor statistically significantly different. Therefore, the small difference in test distance between the Maddox Wing (30 cm) and the other tests (33 cm) was not likely to be important.

A key aim of the current study was to investigate whether differences in the design of heterophoria tests could affect the measured findings through the potential impact on accommodation. Good control of accommodation is thought to be critical when measuring heterophoria in young adults and children.^4^, ^6^, ^7^, ^8^, ^9^, ^23^ For this reason, Pointer^7^ modified the tangent scale of the Maddox Wing test to reduce the font size and added a low spatial frequency square wave grating pattern (two horizontal bars straddling the number scale), ostensibly to provide an optimal stimulus to the accommodative system.^10^, ^11^, ^12^ Pointer reported that the repeatability of the Maddox Wing test was best with the smaller font size and grating scale. However, whether the higher repeatability was due to the presence of the grating or the smaller font was not clear, as the square wave grating did not improve repeatability with the larger (10 pt) target. The results of the present study suggest that the size of the scale font, rather than the addition of the square wave grating, is the more important factor in stabilising accommodation.

Few investigations have directly compared the Howell phoria card, which incorporates the same type of grating stimulus suggested by Pointer,^7^ with other near heterophoria tests. Wong et al.,^9^ in a sample of 72 adults, showed that differences in heterophoria measurements between the Howell card, near von Graefe procedure and Muscle Imbalance Measure (MIM) card (a commercial modification of the Thorington method^24^) methods were small and less than 0.8 Δ. However, they reported better test–retest repeatability with the Howell card than the von Graefe method (but not the MIM card). Their target for the von Graefe method used N5 size print, which is similar in size to the scale on the Howell card but without the additional grating bars, suggesting a potential effect of the bars. On the other hand, the MIM card was similarly repeatable but did not have the additional grating bars, which suggests that the grating bars on the Howell card did not provide any additional stimulus to accommodation, which is consistent with the present results. Better repeatability with the Howell card compared to the von Graefe method has also been reported in other investigations.^25^, ^26^, ^27^

Using a larger sample of 104 adults, Maples and colleagues^23^ compared heterophoria measurements using the von Graefe and Howell card methods. Unlike previous studies,^9^, ^25^, ^26^ a significant difference was found, resulting in, on average, more exophoria with the von Graefe method. The authors suggested that differences in the test protocols may have contributed to the greater exophoria with the von Graefe method as this procedure was carried out using a phoropter. Although not all studies comparing the von Graefe method with a phoropter to the free space Howell card have found greater exophoria,^25^, ^26^ there is evidence that the use of a phoropter may induce more exophoria and poorer repeatability compared with non‐phoropter‐based heterophoria tests.^28^

Facchin and Maffioletti^29^ used a new Thorington (or Prentice) method, similar to the Howell card^9^ and reported normative data when comparing this method with the von Graefe technique. They used a large sample of 315 participants that included both presbyopic and non‐presbyopic individuals. Despite the assumed differences in accommodation between these groups, no significant differences in heterophoria were found (within groups), suggesting that differences in accommodative ability were not significant contributory factors.

### Test–retest repeatability

Test–retest measurements between the heterophoria tests were not significantly different (Table 1). This result is generally consistent with several studies comparing the Howell card with other methods (except for the von Graefe technique),^9^, ^23^, ^25^, ^26^, ^27^ although it should still be interpreted with caution. A larger sample may have included participants with a greater range of heterophoria, thus giving more confidence in the repeatability results. For the same reason, an analysis of the limits of agreement, which is likely to be more informative when considering differences between tests, would not be useful.^30^ However, we have included Bland and Altman plots^30^ comparing differences between test and retest for the two versions of the HHT as well as comparing the HHT Line test with the Howell card as Figure S1. On the other hand, the a priori requirement for the present study was a sample size large enough to detect a difference of 1 Δ between measurements. This value was assumed to be sufficient to conclude that differences in the target configuration on the new HHT cards, namely the inclusion of the grating bars, could be attributed to better accommodative control.

### Accommodation

Not unexpectedly, given that the heterophoria measurements across tests were not significantly different, the measured accommodative responses were also not significantly different (See Table 1). It is well established that the accommodative response, both steady state^11^, ^12^, ^13^ and dynamic,^10^ is optimal for low or medium spatial frequencies. However, it does not necessarily follow that adding a low spatial frequency grating to the heterophoria target would aid accommodation. In general, the targets used to measure heterophoria are likely to be comprised of multiple spatial frequencies and would therefore provide a sufficient stimulus to accommodation for the target distance. Using what they termed naturalistic stimuli, Ciuffreda et al.^31^ measured accommodative responses to a variety of different targets including street maps, telephone directories, magazine and book texts, newspaper print and newspaper cartoons. Despite variations in the composition of the targets in terms of their constituent spatial frequency content, text size, contrast and form, all targets were sufficient to produce an appropriate accommodative response. No significant differences were found in the accommodative responses across the target types.^31^ A corollary to this result is that the average accommodative lag across the target types, which ranged from 0.66D to 0.74D, was also consistent and within the expected range for the age of the sample.^32^

A potentially more important factor in controlling the accommodative response is likely to be the size of the heterophoria scale.^28^ Pointer^7^ reported that the repeatability of the Maddox Wing test was best with the smallest font target (4 pt), albeit with the additional grating bars compared with the larger font size (10 pt). This suggests that the smaller font size was better at activating or stimulating accommodation and is consistent with the reported effect of font size on the subjective amplitude of accommodation.^33^ Given that no differences were found in accommodative responses between the heterophoria targets, as with the heterophoria results, the small difference in test distance between the Maddox Wing (30 cm) and the other tests (33 cm) was unlikely to be important in terms of the accommodative responses.

Although the measurements of accommodative responses were not performed simultaneously with the heterophoria measurements, they were performed under the same conditions (i.e., lighting, test distances, targets and instructions to participants). As a result, it is likely that the resultant accommodative findings represent responses that would occur when heterophoria measurements are performed. In summary, previous work,^7^, ^31^, ^33^ together with the results of the current study, suggests that as long as the heterophoria target contains sufficient detail (i.e., a small letter or number scale), accommodation will respond appropriately and result in consistent heterophoria measurements, at least in a young adult population. The present sample comprised motivated young adults; as such, results may differ in other populations such as children, who may not follow instructions as well or find it more difficult to maintain accurate fixation.

## CONCLUSIONS

The addition of a spatial frequency defined grating pattern (i.e., two horizontal bars subtending 2.5 cpd) to a newly designed Prentice card did not affect the measured heterophoria versus without bars or compared with the Howell card or Maddox Wing test.

Although accommodation responds optimally to low or medium spatial frequencies, there were no significant differences found in accommodative responses between the targets in the Maddox Wing, Howell card or new HHT cards.

The newly designed HHT cards, based on the original Prentice (or Thorington) method,^14^ offer a cost‐effective method to assess near heterophoria comparable to the Maddox Wing and Howell card. The HHT cards (or other variations of the Prentice method^29^) may be used in a young adult population either with or without additional horizontal lines superimposed on the scale. Further work on a larger sample that includes children would be helpful to establish normative data.

## AUTHOR CONTRIBUTIONS

**John Siderov:** Conceptualization (lead); formal analysis (supporting); methodology (equal); writing – original draft (lead); writing – review and editing (equal). **Baskar Theagarayan:** Formal analysis (lead); investigation (equal); methodology (equal); writing – review and editing (equal). **Muneebah Zahir:** Formal analysis (supporting); investigation (equal); writing – review and editing (supporting). **Niall J. Hynes:** Formal analysis (supporting); methodology (equal); writing – review and editing (equal).

## FUNDING INFORMATION

This project was supported with funding from the University of Huddersfield University Research Fund, Award Number URN020‐01.

## CONFLICT OF INTEREST STATEMENT

All authors declare that there are no conflicts of interest.

## Supporting information


Figure S1.

## Data Availability

Reasonable requests for original data may be made to the corresponding author.
