# Supplementary material for: Additional accommodative controls in near heterophoria targets do not improve accommodative responses in young adults
Source: Ophthalmic Physiol Opt. 2025 Feb 25;45(3):752–60. doi: 10.1111/opo.13476 (PMC11976503; doi:10.1111/opo.13476)
Supplement: Supplementary file 1 — Figure S1. [file OPO-45-752-s001.docx]

**APPENDIX**

Supplementary Figure 1. Bland and Altman plots depicting, in the top panel, the differences in mean heterophoria (Δ) between the two versions of the Huddersfield Heterophoria Test (HTT and HTT Line) against their average. Similarly, in the bottom panel, the differences in mean heterophoria (Δ) between the HTT Line test and the Howell card against their average are shown (retest condition). The central dotted lines in each panel represent the mean differences, while the dashed lines represent the 95% limits of agreement. The error bars shown in each panel represent the 95% confidence intervals for the limits of agreement. The relatively small sample size makes interpretation of these plots difficult.
